# Knowledge Gaps, Treatment Preferences, and Unmet Clinical Needs Among Patients With Inflammatory Bowel Disease: A Cross‐Sectional Study

**DOI:** 10.1002/kjm2.70248

**Published:** 2026-06-10

**Authors:** Yi‐Chen Wu, Chien‐Ming Chen, Tony Kou, Tai‐Di Chen, Ming‐Jung Meng, Chen‐Wang Chang, Jen‐Wei Chou, Tien‐Yu Huang, Cheng‐Tang Chiu, Yu‐Bin Pan, Puo‐Hsien Le, Ming‐Yao Su

**Affiliations:** ^1^ College of Medicine Chang Gung University Taoyuan City Taiwan; ^2^ Department of Medical Imaging and Interventions Chang Gung Memorial Hospital at Linkou Taoyuan Taiwan; ^3^ Chang Gung Inflammatory Bowel Disease Center Taoyuan Taiwan; ^4^ Department of Gastroenterology and Hepatology Chang Gung Memorial Hospital at Linkou Taoyuan Taiwan; ^5^ Department of Anatomic Pathology Chang Gung Memorial Hospital Taoyuan Taiwan; ^6^ School of Medicine National Tsing Hua University Hsinchu Taiwan; ^7^ Taiwan Association for the Study of Intestinal Diseases (TASID) Taoyuan Taiwan; ^8^ Division of Gastroenterology, Department of Internal Medicine MacKay Memorial Hospital Taipei Taiwan; ^9^ MacKay Junior College of Medicine, Nursing and Management Taipei Taiwan; ^10^ MacKay Medical College New Taipei City Taiwan; ^11^ Center for Digestive Medicine, Department of Internal Medicine China Medical University Hospital Taichung Taiwan; ^12^ School of Chinese Medicine China Medical University Taichung Taiwan; ^13^ Division of Gastroenterology and Hepatology, Department of Internal Medicine Tri‐Service General Hospital, National Defense Medical Center Taipei Taiwan; ^14^ Chang Gung Microbiota Therapy Center Taoyuan Taiwan; ^15^ Biostatistical Section, Clinical Trial Center Chang Gung Memorial Hospital at Linkou Taoyuan Taiwan; ^16^ Division of Gastroenterology and Hepatology, Department of Internal Medicine New Taipei Municipal Tucheng Hospital New Taipei City Taiwan

**Keywords:** advanced therapy, IBD‐KNOW, steroids, treatment preferences, unmet needs

## Abstract

This study utilized a cross‐sectional survey via a structured questionnaire to evaluate disease‐related knowledge, treatment preferences, and unmet needs among 200 adults with confirmed inflammatory bowel disease (IBD) at a tertiary referral center. The questionnaire specifically assessed disease knowledge using the 24‐item IBD‐KNOW score, priorities for treatment attributes, preferred administration modalities, and self‐reported unmet needs. Results indicated a mean IBD‐KNOW score of 14.8/24, reflecting moderate overall knowledge, with female sex identified as an independent predictor of higher knowledge levels (OR: 1.87, *p* = 0.044). In terms of treatment, patients prioritized efficacy (8.9/10) over safety (8.5) and convenience (8.1); notably, 49% ranked efficacy as the most important factor, whereas 88% ranked convenience as the least important. The most preferred administration modalities were once‐daily oral therapy (31%) and bimonthly subcutaneous injection (29.5%). While 43.5% of respondents reported no unmet needs, significant concerns persisted regarding the fear of disease progression (24.5%), medication cost (22.5%), and long waiting times for care (20.5%). These findings suggest that although IBD patients possess moderate disease‐specific knowledge and place a strong emphasis on treatment efficacy and long‐interval regimens, clinical focus is still needed to address ongoing anxieties regarding long‐term outcomes, financial burdens, and healthcare access.

## 1 Introduction

Inflammatory bowel disease (IBD) is a chronic, relapsing inflammatory disorder of the gastrointestinal tract, affecting more than 10 million people worldwide [1]. Beyond persistent gastrointestinal symptoms, IBD disrupts daily routines, impairs work productivity, and imposes substantial psychosocial burdens on patients [2, 3, 4, 5]. Despite therapeutic advances, long‐term management remains challenging. Optimal outcomes increasingly depend on patient engagement, shared planning, and a holistic care model that addresses not only clinical control but also quality of life, emotional well‐being, and social functioning [6].

Shared decision‐making (SDM) has therefore become a central component of chronic disease management, including IBD. Effective SDM requires that patients be adequately informed about their disease and available treatment options, and feel supported in expressing their values and preferences [7]. However, existing studies show that many patients lack sufficient disease‐related knowledge and access to clear educational resources, limiting their ability to participate meaningfully in treatment decisions [8]. To quantify patient understanding, the 24‐item IBD‐KNOW questionnaire has been developed and validated and has been applied in several regions—including Korea and the United States—revealing notable geographic and cultural variation in IBD‐related knowledge [9, 10]. Assessing knowledge within local populations is therefore essential to designing appropriate educational strategies and improving SDM.

International surveys further highlight gaps in current IBD care, particularly regarding long‐term treatment effectiveness, individualized therapy, and psychosocial support [6, 11, 12]. Treatment satisfaction depends not only on clinical efficacy but also on patients' preferences regarding medication modes, dosing intervals, and long‐term expectations [11]. At the same time, unmet needs—such as treatment costs, difficulties accessing care, limited communication, and insufficient lifestyle or psychological support—continue to affect patient experience and outcomes [8, 12]. Understanding what patients value and where existing care falls short is fundamental to advancing truly patient‐centered IBD management.

Despite growing interest in patient‐centered approaches, region‐specific data in Asia remain limited. No comprehensive study has evaluated IBD patients' disease knowledge, treatment preferences, and unmet needs in this setting. To address this gap, we assessed disease‐related knowledge using the IBD‐KNOW tool, characterized priorities for treatment attributes, and identified patient‐reported unmet needs. These findings aim to inform culturally relevant strategies that support SDM and improve holistic IBD care.

## 2 Material and Methods

### 2.1 Study Design and Population

We conducted a single‐center cross‐sectional study at Linkou Chang Gung Memorial Hospital in Taiwan to evaluate disease knowledge, treatment preferences, and unmet needs among patients with IBD. The protocol adhered to the ethical principles of the 1975 Declaration of Helsinki (6th revision, 2008) and was approved by the institutional review board of Chang Gung Memorial Hospital (Approval No. 202500772B0). Adults with a confirmed diagnosis of ulcerative colitis (UC) or Crohn's disease (CD) were eligible; there was no upper age limit. Patients with insufficient clinical records were excluded. Approximately 200 eligible patients diagnosed on or before September 30, 2024, were identified. All participants provided written informed consent and completed the survey once. Questionnaire responses and clinical data were collected between September 22 and October 22, 2025.

### 2.2 Survey Instrument and Data Collection

A structured, self‐administered questionnaire captured demographics, lifestyle factors, diagnostic history, treatment experiences, and patient priorities. Regarding diagnostic history, the number of medical consultations prior to a definitive diagnosis was recorded. Here, a “physician” was defined as any medical doctor—including community‐based general practitioners, internists at regional hospitals, and specialists (such as gastroenterologists or colorectal surgeons) at tertiary centers—consulted for IBD‐related symptoms. Disease‐specific knowledge was assessed using the validated 24‐item IBD‐KNOW questionnaire, which yields a total score from 0 to 24 [10]. For this study, the original English instrument was translated into Chinese by bilingual clinicians experienced in IBD care to ensure conceptual equivalence; however, formal psychometric validation of this translation has not yet been conducted. A supplemental question regarding the impact of smoking on UC (“Smoking worsens ulcerative colitis”) was analyzed independently and excluded from the total score to maintain the integrity of the original instrument. Treatment priorities were evaluated by asking patients to rate the importance of treatment‐related domains (including effectiveness, safety, convenience, and aspects of daily functioning) on a 0–10 scale. To explore unmet needs, patients were presented with 26 items derived from previous work on IBD care gaps [12], covering concerns such as disease progression, medication costs, and communication issues. Participants could select multiple applicable items and an option indicating no unmet needs. This allowed estimation of the prevalence of each concern within the cohort.

### 2.3 Statistical Analysis

Analyses were performed using IBM SPSS Statistics for Windows, version 30.0 (IBM Corp., Armonk, NY, USA). Baseline characteristics were summarized as means with standard deviations (SD) for continuous variables and as frequencies (percentages) for categorical variables. Differences between UC and CD were assessed with independent‐sample *t‐*tests for continuous variables and Chi‐squared tests or Fisher's exact tests for categorical variables. For polychotomous variables (categorical variables with multiple levels), an overall *p*‐value was first calculated. In cases where the overall test yielded significant results, post hoc pairwise comparisons were conducted, and a Bonferroni correction was applied to adjust the significance threshold. Additionally, the Mann–Whitney *U* test was employed to compare the preference rankings (efficacy, safety, and convenience) between patients with and without prior experience in advanced therapies to assess potential experience‐based bias. A two‐tailed *p*‐value < 0.05 (or the adjusted threshold for post hoc tests) was considered statistically significant.

To identify factors associated with higher disease knowledge, IBD‐KNOW scores were dichotomized using the cohort median as the cutoff (high vs. lower knowledge). Univariate logistic regression was first applied to candidate predictors, including IBD type, gender, age, age at diagnosis, disease duration, diagnostic delay, number of physicians at diagnosis, lifestyle habits (smoking, alcohol, and betel nut use), clinical history (hospitalizations or emergency visits in the past year), and experience with advanced therapies. Variables with *p* < 0.10 were entered a multivariate logistic regression model to determine independent predictors. Odds ratios (ORs) with 95% confidence intervals (CIs) were calculated. To explore the correlation between disease knowledge and patient perspectives, unmet needs and preferred administration routes were compared between the high and lower knowledge groups. Fisher's exact tests were used for individual unmet need items. For the overall distribution of preferred administration routes, the Fisher–Freeman–Halton exact test was employed, with post hoc analysis performed using adjusted residuals (absolute values > 1.96 indicated statistical significance). Missing data were not imputed; cases with missing values were excluded from the relevant analyses.

## 3 Results

### 3.1 Baseline Patient Characteristics

Of 227 returned questionnaires, 200 were complete and included in the analysis (108 UC, 92 CD). Baseline characteristics are summarized in Table 1. The cohort was predominantly male (66.5%), with similar sex distribution in UC and CD (66.7% vs. 66.3%, *p* = 0.957). Mean age was 43.0 ± 14.1 years; UC patients were older than CD patients (45.6 ± 13.0 vs. 40.0 ± 14.7 years, *p* = 0.004). Age at diagnosis tended to be higher in UC than CD (39.2 vs. 35.5 years, *p* = 0.057). Mean disease duration was 5.52 ± 6.59 years and was numerically longer in UC than CD (6.39 vs. 4.50 years, *p* = 0.088). Diagnostic delay was similar between groups (both ~1.7 years, *p* = 0.761). CD patients were more likely to have consulted ≥ 5 physicians before diagnosis (17.4% vs. 4.6%, *p* = 0.015), suggesting a more complex diagnostic pathway.

**TABLE 1:** Comparison of demographic and clinical characteristics between patients with ulcerative colitis (UC) and Crohn's disease (CD).

	Overall (*n* = 200)	UC (*n* = 108)	CD (*n* = 92)	*p*	Bonferroni *p*
Baseline data					
Gender (male)	133 (66.50%)	72 (66.67%)	61 (66.30%)	0.957	
Age (mean ± SD years)	43.0 ± 14.1	45.60 ± 13.04	39.97 ± 14.67	0.004*	
Age at diagnosis (mean ± SD years)	37.49 ± 13.90	39.21 ± 12.90	35.47 ± 14.80	0.057	
Disease duration (mean ± SD years)	5.52 ± 6.59	6.39 ± 7.43	4.50 ± 5.29	0.088	
Diagnostic delay (mean ± SD years)	1.68 ± 3.60	1.64 ± 4.10	1.72 ± 3.60	0.761	
Diagnosis made by *N*th physician				0.039*	
1	60 (30.00%)	33 (30.56%)	27 (29.35%)		1.000
2	69 (34.50%)	39 (36.11%)	30 (32.61%)		1.000
3	38 (19.00%)	25 (23.15%)	13 (14.13%)		0.525
4	12 (6.00%)	6 (5.56%)	6 (6.52%)		1.000
≥ 5	21 (10.50%)	5 (4.63%)	16 (17.39%)		0.015*
Smoking status				0.010*	
Never	129 (64.50%)	70 (64.81%)	59 (64.13%)		1.000
Past smoker	51 (25.50%)	33 (30.56%)	18 (19.57%)		0.228
Current smoker	20 (10.00%)	5 (4.63%)	15 (16.30%)		0.018*
Alcoholism				0.411	
Never	138 (69.00%)	72 (66.67%)	66 (71.74%)		
Past drinker	43 (21.50%)	23 (21.30%)	20 (21.74%)		
Current drinker	19 (9.50%)	13 (12.04%)	6 (6.52%)		
Betel nut chewing				0.332	
Never	178 (89.00%)	99 (91.67%)	79 (85.87%)		
Past chewer	18 (9.00%)	8 (7.41%)	10 (10.87%)		
Current chewer	4 (2.00%)	1 (0.93%)	3 (3.26%)		
Hospitalizations in the past year (mean ± SD times)	0.76 ± 1.31	0.66 ± 1.37	0.88 ± 1.24	0.232	
Emergency visits in the past year (mean ± SD times)	0.54 ± 0.99	0.41 ± 0.87	0.70 ± 1.10	0.043*	
Advance therapy experience (yes)	173 (86.5%)	89 (82.41%)	84 (91.30%)	0.066	
Prolonged steroid exposure (yes)	105 (52.5%)	53 (49.07%)	52 (56.52%)	0.293	

*Note:* Values are presented as mean ± standard deviation (SD) for continuous variables and as number (percentage) for categorical variables. *p*‐values for continuous variables were calculated using independent‐sample *t*‐tests. Overall *p*‐values for categorical variables were calculated using Pearson's Chi‐squared test or Fisher's exact test. For polychotomous variables with a significant overall *p*‐value (*p* < 0.05), “Bonferroni *p*‐value” represents the adjusted *p*‐value for pairwise comparisons between the UC and CD groups. The significance threshold was adjusted using the Bonferroni correction to maintain the family‐wise error rate.

Abbreviations: CD, Crohn's disease; CI, confidence interval; IBD‐KNOW, Inflammatory Bowel Disease Knowledge; OR, odds ratio; UC, ulcerative colitis.

* *p* < 0.05 (or adjusted *p* < 0.05 for post hoc tests) was considered statistically significant.

Current smoking was more frequent in CD than UC (16.3% vs. 4.6%, *p* = 0.018), whereas past and never‐smoker status did not differ significantly. Alcohol use and betel nut chewing were uncommon overall and comparable between groups. Regarding healthcare utilization, CD patients had more IBD‐related emergency department visits in the preceding year than UC patients (0.70 ± 1.10 vs. 0.41 ± 0.87, *p* = 0.043), while hospitalization frequency and prior exposure to advanced therapies were slightly higher in CD but did not reach statistical significance.

### 3.2 Disease‐Related Knowledge

#### 3.2.1 Knowledge Scores and Commonly Understood Concepts

IBD‐KNOW scores ranged from 0 to 24 (mean 14.8 ± 5.2; median 15), indicating moderate knowledge with wide inter‐individual variability. The top quartile achieved a mean score of 20.9, representing a highly informed subgroup. Items most frequently answered correctly related to general disease awareness and basic clinical facts. High proportions of patients knew that dietary restrictions are recommended in IBD (95.0%) and that intestinal inflammation may persist despite symptom improvement (92.0%). Many also recognized the risk of anemia due to chronic intestinal inflammation (81.5%), basic gastrointestinal anatomy (78.0%), the contribution of family history (76.0%), the harms of long‐term steroid use (74.5%), the detrimental effect of smoking in CD (74.0%), typical locations of CD (71.5%), and the need for colorectal cancer surveillance (65.5%).

A supplementary item—“Smoking worsens ulcerative colitis”—was added to explore understanding of the divergent effects of smoking in UC and CD. Whereas 74.0% correctly agreed that smoking cessation is important to prevent worsening of CD (original IBD‐KNOW item), only 5.5% correctly identified the UC statement as false. This marked discrepancy highlights a substantial knowledge gap regarding disease‐specific effects of smoking and suggests that generic anti‐smoking messages may overshadow more nuanced information.

#### 3.2.2 Predictors of Higher Knowledge

Using the median IBD‐KNOW score as a cutoff, 91 patients (45.5%) were classified as having high knowledge. Logistic regression results are shown in Table 2. In univariate analyses, consulting > 3 physicians before diagnosis was associated with higher knowledge (OR: 2.43, *p* = 0.025), and female sex showed a borderline association (OR: 1.80, *p* = 0.051). In the multivariate model, female sex remained the only independent predictor of high knowledge (adjusted OR: 1.87, *p* = 0.044), whereas the association with having seen > 3 physicians narrowly missed significance (adjusted OR: 2.21, *p* = 0.051).

**TABLE 2:** Univariate and multivariate logistic regression analyses of predictors associated with high IBD‐KNOW scores.

Variables	Patients with high IBD‐KNOW score (*n* = 91)	Univariate analysis		Multivariate analysis	
		OR (95% CI)	*p*	OR (95% CI)	*p*
IBD type					
CD	47 (51.69%)	1.519 (0.867–2.662)	0.144		
Gender					
Female	37 (40.67%)	1.804 (0.997–3.265)	0.051	1.867 (1.016–3.431)	0.044*
Age (year)	91 (100.00%)	0.988 (0.969–1.008)	0.254		
Age at diagnosis (year)	91 (100.00%)	0.983 (0.962–1.003)	0.094	0.985 (0.964–1.006)	0.154
Disease duration (year)	91 (100.00%)	1.033 (0.995–1.073)	0.091	1.026 (0.987–1.066)	0.194
Diagnostic delay (year)	91 (100.00%)	1.039 (0.972–1.110)	0.259		
Physician number at diagnosis					
> 3	21 (23.08%)	2.425 (1.119–5.253)	0.025*	2.205 (0.995–4.888)	0.051
Smoking status					
Current smoker	8 (8.79%)	0.779 (0.304–1.997)	0.603		
Alcoholism					
Current user	11 (12.09%)	1.736 (0.667–4.519)	0.259		
Betel nut chewing					
Current chewer	2 (2.20%)	1.202 (0.166–8.708)	0.855		
Hospitalizations in the past year	91 (100.00%)	1.168 (0.933–1.463)	0.175		
Emergency visits in the past year	91 (100.00%)	1.039 (0.784–1.378)	0.789		
Advance therapy experience					
Yes	80 (87.91%)	1.251 (0.549–2.852)	0.594		

*Note:* Values represent odds ratios (ORs) with 95% confidence intervals (CIs) and corresponding *p*‐values. High IBD‐KNOW score was defined as a score greater than the cohort median. Variables with a *p*‐value < 0.10 in the univariate analysis were included in the multivariable logistic regression model. Reference groups for categorical variables: male (gender), UC (IBD type), ≤ 3 (physician number), never/former user (smoking status, alcoholism, and betel nut chewing), and no (advanced therapy experience).

Abbreviations: CD, Crohn's disease; CI, confidence interval; IBD‐KNOW, Inflammatory Bowel Disease Knowledge; OR, odds ratio; UC, ulcerative colitis.

* *p* < 0.05 was considered statistically significant.

#### 3.2.3 Correlation of Knowledge With Unmet Needs and Treatment Preferences

Significant differences in unmet needs and treatment preferences were observed between the two knowledge groups. Regarding unmet needs, the high‐knowledge group reported significantly higher concerns regarding body image (9.9% vs. 1.8%, *p* = 0.025), lack of psychological support (6.6% vs. 0.9%, *p* = 0.048), and interest in Traditional Chinese Medicine integration (13.2% vs. 3.7%, *p* = 0.018). Furthermore, the preferred administration routes differed significantly between groups (*p* = 0.013). Post hoc analysis revealed that high‐knowledge patients demonstrated a significant preference for on‐body injectors (adjusted residual = 3.0), whereas low‐knowledge patients were significantly more likely to prefer intravenous injection (adjusted residual = 2.5).

### 3.3 Treatment Priorities and Attributes

#### 3.3.1 Effectiveness, Safety, and Convenience

**FIGURE 1:**
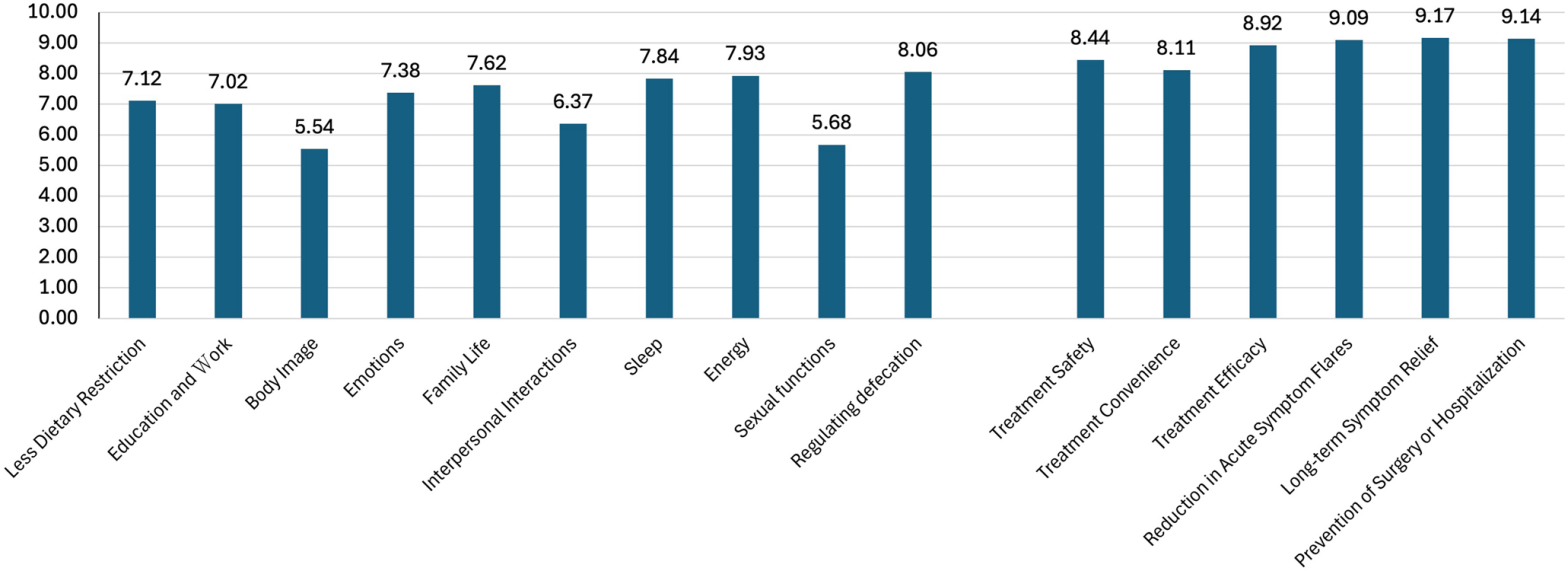
Patient‐reported priorities in quality of life domains and treatment outcomes. Mean importance scores (0–10 scale) for 16 treatment‐related aspects, including 10 quality of life domains (adapted from the IBD Disk and IBD‐Bx frameworks), three core treatment attributes (effectiveness, safety, convenience), and three key clinical outcomes. Long‐term remission received the highest rating (9.17), followed by avoidance of hospitalization or surgery (9.14) and reduction of acute flares (9.09). IBD, inflammatory bowel disease; IBD‐Bx, Inflammatory Bowel Disease‐related Behaviors questionnaire.

Patients rated 13 treatment‐related domains on a 0–10 importance scale, including three core attributes—effectiveness, safety, and convenience—adapted from the IBD‐Bx and IBD Disk instruments [13, 14]. Effectiveness received the highest mean rating (8.92), followed by safety (8.45) and convenience (8.11) (Figure 1), indicating that disease control is the predominant concern, with safety and practicality secondary but still important.

#### 3.3.2 Daily Functioning and Quality of Life

Among domains reflecting daily life, the ability to regulate bowel habits had the highest mean importance (8.06), followed by energy levels (7.93), and sleep quality (7.84). Family and social life (7.62) and emotional well‐being (7.38) were also highly valued. In contrast, body image (5.54) and sexual function (5.68) received lower average ratings, although these issues remain clinically relevant for some patients. Overall, the pattern underscores the importance placed on maintaining daily functioning, relationships, and psychological health alongside symptom control (Figure 1).

#### 3.3.3 Critical Treatment Outcomes

When asked about key outcomes, patients rated reducing acute flares, maintaining long‐term remission, and avoiding surgery or hospitalization as extremely important, with mean scores above 9.0 for all three (9.09, 9.17, and 9.11, respectively; Figure 1). These results highlight patients' strong preference for durable disease control and avoidance of invasive interventions.

### 3.4 Attitudes Toward Steroids and Advanced Therapies

#### 3.4.1 Steroid Use and Patient Concerns

Overall, 149 of 200 patients (74.5%) reported concern about corticosteroid use, while 51 (25.5%) were not particularly worried. A history of prolonged steroid exposure (> 3 consecutive months) was reported by 105 patients (52.5%); 49 (46.7%) had such exposure within the prior year and 56 (53.3%) more than 1 year earlier. These findings indicate that extended steroid use remains common and that most patients are wary of its adverse effects, which include increased risks of infection, osteoporosis, diabetes, and cardiovascular disease [15]. The results support the need for steroid‐sparing strategies and clear communication about tapering and alternative therapies.

#### 3.4.2 Prioritization of Efficacy, Safety, and Convenience

**FIGURE 2:**
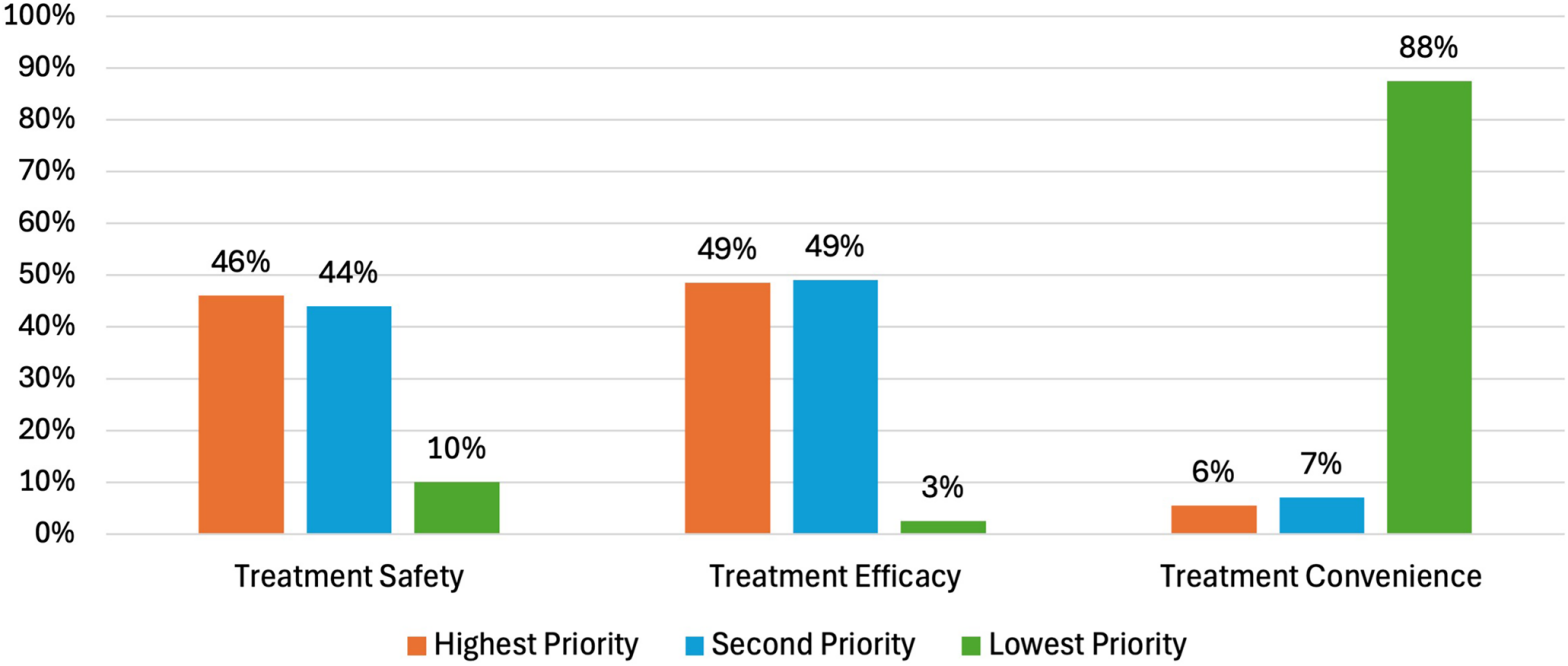
Patient prioritization of efficacy, safety, and convenience in advanced therapy decision‐making. Ranking distributions for three core treatment attributes among 200 patients. Nearly half (49%) ranked efficacy as the top priority, whereas convenience was ranked lowest by 88%, indicating that patients place markedly greater emphasis on clinical effectiveness and safety over logistical convenience.

When asked to rank efficacy, safety, and convenience for advanced therapies, 49% of patients rated efficacy as their highest priority and only 3% as lowest. Safety was ranked first by 46% and second by 44% of respondents. In contrast, convenience was ranked lowest by 88% of patients and first by only 6% (Figure 2). Thus, most patients prioritized maximizing disease control and minimizing serious adverse effects over logistical convenience.

To address potential bias regarding treatment requirements, a subgroup analysis was performed comparing patients with prior advanced therapy experience (*n* = 173) and those who were treatment‐naive (*n* = 27). The Mann–Whitney *U* test revealed no statistically significant differences between the two groups in the priority ranking of treatment safety (*p* = 0.176), efficacy (*p* = 0.616), and convenience (*p* = 0.283). Both subgroups consistently prioritized efficacy and safety over logistical convenience.

#### 3.4.3 Preferred Treatment Modalities

**FIGURE 3:**
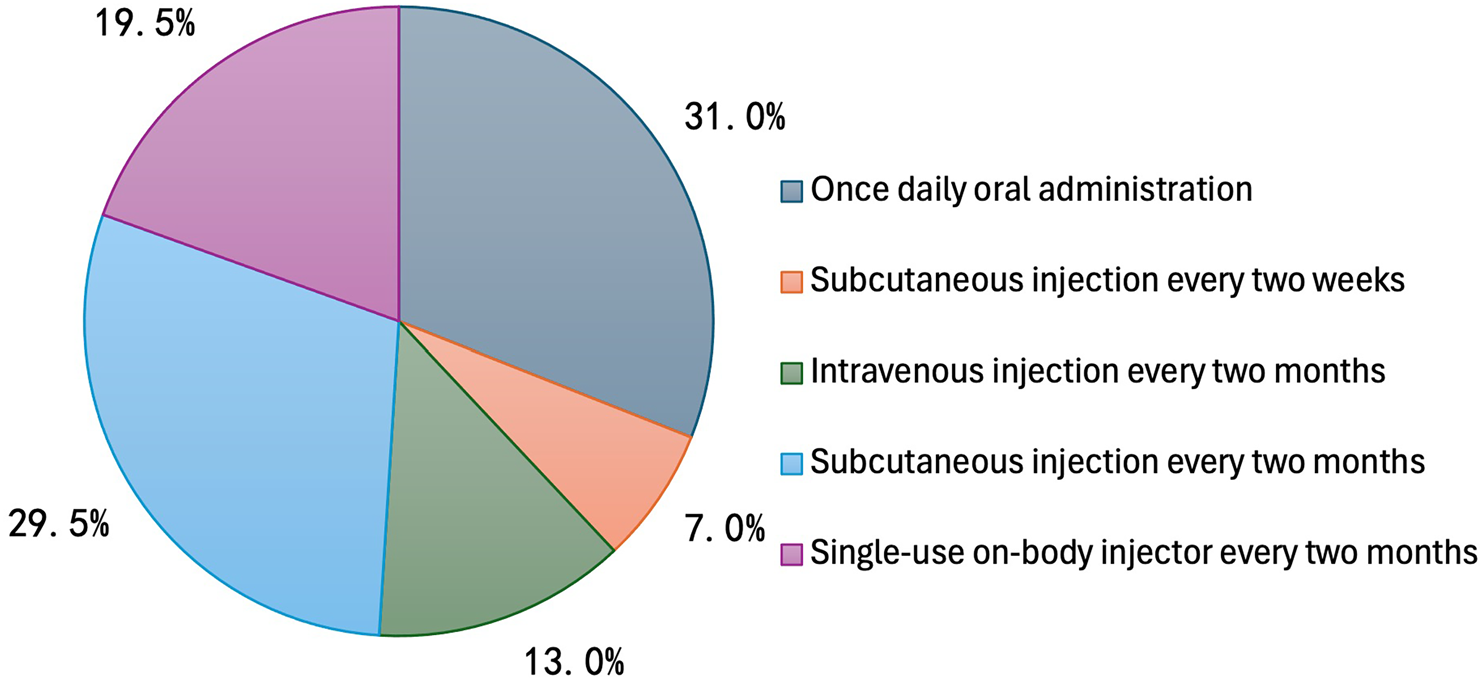
Patient preferences for drug administration mode and dosing frequency. Preferred routes and frequencies of advanced therapy administration, assuming equivalent efficacy and safety. The most favored options were once‐daily oral medication (31.0%) and subcutaneous injection every 2 months (29.5%). On‐body injector systems were preferred by 19.5%, while biweekly subcutaneous injection was least preferred (7.0%).

Assuming equivalent efficacy and safety, patients then chose their preferred administration modality. Once‐daily oral medication was most frequently selected (31.0%), followed by subcutaneous injection every 2 months (29.5%). Single‐use on‐body injector devices were preferred by 19.5%, intravenous infusion every 2 months by 13.0%, and biweekly subcutaneous injection by 7.0% (Figure 3). These preferences indicate that, although oral therapy is attractive, many patients favor infrequent, low‐burden injection schedules over more frequent dosing.

Sub‐group analysis revealed a significant difference in preferences between therapy‐naïve patients and those with experience in advanced therapy (*x* ^2^ = 12.604, df = 4, *p* = 0.013). Specifically, the conventional therapy group showed a significantly higher preference for the “once‐daily oral” route compared to the advanced therapy group (59.3% vs. 26.6%, *p* < 0.05 after Bonferroni adjustment). Conversely, patients with experience in advanced therapy demonstrated a more diverse distribution across long‐acting injectable options.

### 3.5 Patient‐Reported Unmet Needs

**FIGURE 4:**
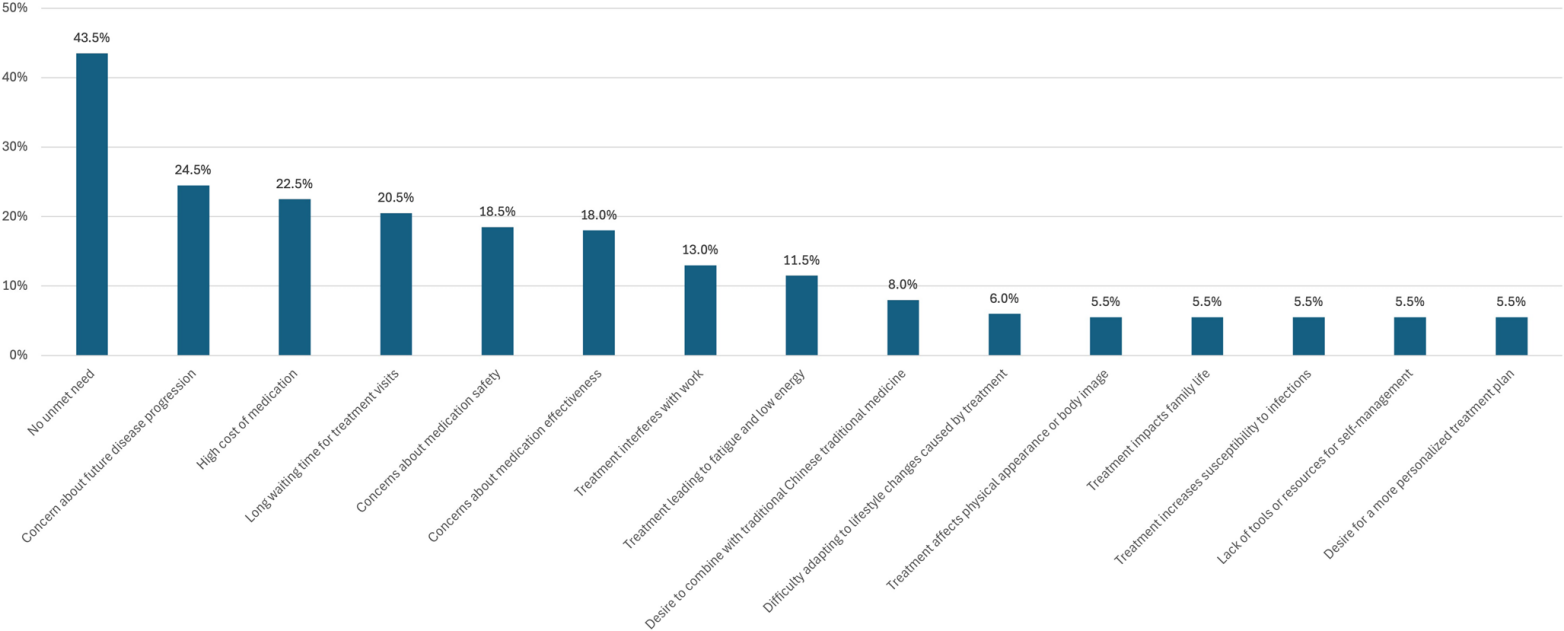
Prevalence of patient‐identified unmet needs in IBD care. Proportion of patients endorsing each of 26 predefined unmet needs. The most frequently reported concerns were fear of future disease progression (24.5%), high medication costs (22.5%), and long wait times for care (20.5%). Notably, 43.5% of patients reported no current unmet needs.

Participants could select from 26 potential unmet needs plus an option for “no unmet needs.” Overall, 87 patients (43.5%) reported no current unmet need, whereas 113 (56.5%) endorsed at least one concern (Figure 4). The most common unmet needs were worry about future disease progression (24.5%), high medication costs (22.5%), and long waiting times for medical care (20.5%), reflecting anxiety about long‐term outcomes, financial burden, and access to services [16]. Concerns about medication safety and long‐term risks were reported by 18.5% of patients, and 18.0% felt that treatment effectiveness was insufficient. Thirteen percent indicated that treatment interfered with work, and 11.5% reported fatigue or low energy related to treatment.

A total of 8.0% of respondents expressed interest in integrating TCM into their care, highlighting a culturally specific preference for complementary approaches [17]. Other unmet needs, each endorsed by 5%–6% of patients, included body‐image concerns, impact on family or social life, fear of infections, lack of helpful educational or self‐management tools, and desire for more personalized treatment plans.

Taken together, more than half of patients reported at least one unmet need, most commonly fears about disease progression, cost and access barriers, and concerns regarding treatment safety and efficacy. Issues related to work, energy, and cultural preferences further emphasize the importance of multidisciplinary, patient‐centered care that addresses clinical, psychosocial, and logistical dimensions of living with IBD.

## 4 Discussion

The management of IBD has evolved substantially with the introduction of biologics, small molecules, and SDM frameworks, yet important gaps remain in how patients perceive their disease, prioritize treatment attributes, and experience unmet needs—particularly in non‐Western settings. This study provides one of the first large‐scale, patient‐reported assessments of disease knowledge, treatment preferences, and perceived barriers among Taiwanese patients with IBD.

Patients demonstrated a moderate level of disease knowledge (mean IBD‐KNOW score 14.8). Within this cohort, female sex independently predicted higher knowledge. These findings are consistent with reports that female IBD patients may engage more actively in health information‐seeking and education [18, 19]. This disparity suggests a broader “gender gap” in health information behaviors, where female patients often demonstrate a higher disposition to be well‐informed as a proactive self‐management strategy [20]. Beyond clinical consultations, women are more likely to utilize diverse information channels—including digital health platforms, patient associations, and informal social networks—to navigate the complexities of their disease [21]. Our findings parallel previous large‐scale studies, which identified male sex as a consistent predictor of lower disease‐specific knowledge [19]. These patterns suggest that sex‐specific educational outreach, particularly strategies designed to engage male patients who may seek information less spontaneously, could help bridge knowledge gaps and enhance the SDM process.

Beyond demographics, higher disease knowledge correlates with increased concerns regarding body image and psychological support. This “knowledge‐need” paradox suggests that improved health literacy shifts patient focus from symptom control toward holistic quality of life, necessitating clinical attention to the psychosocial burdens of well‐informed patients. Notably, the mean IBD‐KNOW score of 14.8 is comparable to reports from the United States population (14.8) and numerically higher than the previously reported Korean data (11.3) [9]. This trend is even more pronounced among the top 25% of scorers, where our cohort achieved a mean of 20.9, exceeding both the US (19.9) and Korean (17.6) subgroups. While the lack of raw data in earlier reports precludes formal statistical comparison, this trend likely reflects several systemic factors within Taiwan's healthcare landscape. First, the widespread implementation of multidisciplinary teams (MDT) and dedicated IBD case managers in tertiary centers provides patients with continuous, structured education and real‐time consultation via digital platforms like LINE. Second, professional and patient organizations have played a pivotal role in knowledge dissemination. The Taiwan Association for the Study of Intestinal Diseases (TASID) has standardized care through robust physician education and academic seminars, while the Taiwan Intestinal Health Association (TIHA) has been instrumental in organizing patient support groups and large‐scale health education activities. Furthermore, the National Health Insurance (NHI) system's “Chronic Disease Prescription” system ensures frequent touchpoints with healthcare providers, facilitating the repeated reinforcement of disease‐related information. Finally, the temporal gap between our study and earlier cohort likely reflects a global increase in disease awareness and the proliferation of active online patient support communities.

Our study revealed that diagnostic delay was comparable between the two IBD subtypes, with a mean delay of 1.64 ± 4.10 years for UC and 1.72 ± 3.60 years for CD. This finding presents a notable departure from Western literature, where CD typically involves a significantly longer diagnostic interval due to its complex, transmural nature [22, 23]. Furthermore, our observed delay for UC is considerably longer than the global median of 3.2–3.7 months reported in high‐income countries [24]. This unexpected prolongation likely reflects a complex interplay between clinical presentation and local health‐seeking behaviors. Several factors may contribute to this: first, the overlap of early UC symptoms with common benign conditions, such as hemorrhoids or irritable bowel syndrome, often leads to initial under‐recognition in primary care settings. Second, the indolent nature of early UC may reduce the perceived urgency for investigation compared to the alarming systemic features of CD. Finally, the high accessibility of NHI system, in the absence of a mandatory gatekeeper, may paradoxically facilitate “medical drifting” or “doctor‐shopping,” where patients cycle through multiple clinics or prioritize alternative therapies before reaching an IBD specialist.

The extremely low correct response rate to the supplemental item “Smoking worsens ulcerative colitis” (5.5%) compared with the original smoking‐related CD item (74.0%) highlights a specific gap regarding the divergent effects of smoking in UC versus CD. Although smoking is clearly harmful in general [25] and detrimental in CD [26], several analysts suggest a paradoxical protective association in UC [27, 28]. However, a notable “knowledge‐action gap” was observed in our cohort: while 74.0% of patients correctly identified smoking cessation as crucial for preventing CD worsening, the prevalence of current smoking remained significantly higher in CD than in UC patients (16.3% vs. 4.6%, *p* = 0.018).

This discrepancy suggests that awareness of general harm does not inherently translate into behavioral change. Although IBD patients acknowledge general risks, many remain unaware of CD‐specific complications, such as increased flare‐ups, surgical requirements, and postoperative recurrence [29]. Since education alone is often insufficient, effective cessation requires active therapeutic intervention [30]. Beyond knowledge gaps, nicotine dependence serves as a significant barrier, often functioning as a maladaptive coping mechanism for the chronic psychological stress and anxiety associated with IBD. Notably, CD patients exhibit higher relapse rates post‐quitting compared to UC patients, which aligns with the higher smoking prevalence and lower cessation success observed in our CD subgroup [31]. These findings highlight the need for a multidisciplinary approach that integrates smoking cessation clinics and psychiatric support into standard IBD care.

When considering advanced therapies, patients prioritized efficacy, followed by safety, with convenience a distant third—aligning with previous work showing effectiveness as the primary determinant of treatment choice [6, 32]. Our findings parallel multi‐regional DCE and survey data indicating broad preference for strong long‐term efficacy with oral or infrequent injectable administration rather than convenience alone [33, 34]. Crucially, subgroup analysis revealed these priorities remained consistent regardless of prior exposure to advanced therapies. This suggests that the high value placed on clinical outcomes and safety is a fundamental expectation rather than a preference acquired only after initiating such treatments.

Interestingly, our sub‐group analysis showed that patients with prior experience in advanced therapy were significantly less likely to prefer daily oral medications than treatment‐naïve patients. This shift suggests that exposure to the high efficacy of injectable biologics may reduce the initial “pill‐preference,” as patients become more accustomed to—and perhaps prioritize—the clinical benefits and infrequent dosing schedules of advanced treatments over the convenience of oral administration.

Furthermore, the significant correlation between knowledge levels and administration preferences highlights the role of health literacy in treatment decision‐making. The preference for on‐body injectors among high‐knowledge patients suggests a priority for treatment autonomy and the convenience of advanced, home‐based technologies. Conversely, the preference for intravenous administration in the lower‐knowledge group may reflect a higher comfort level with provider‐supervised settings or a lack of confidence in self‐administration. These findings indicate that patient education should not only cover disease facts but also aim to empower patients to navigate diverse therapeutic modalities.

Corticosteroid use was a major concern. Nearly three‐quarters of patients expressed worry, and more than half reported prolonged exposure. These findings mirror prior reports of anxiety about steroid‐related complications [15, 35] and patient ambivalence toward their use [36]. Limited information or restricted access to alternatives may contribute to these concerns [37]. Clear tapering strategies and earlier transition to maintenance therapies may help build trust and support long‐term control.

Patients rated classic clinical outcomes—sustained remission, avoidance of flares, and prevention of surgery—as extremely important, but also valued sleep, energy, emotional well‐being, and family life, consistent with evidence that IBD affects social and psychological functioning [38]. Lower mean scores for sexual function and body image indicate variability in relevance but do not diminish their importance when present [37].

More than half of patients reported at least one unmet need, most commonly fear of disease progression, medication costs, and long waiting times—patterns also seen in European cohorts [12]. Interest in integrating TCM (8%) reflects culturally influenced expectations and supports consideration of integrative, evidence‐based approaches [17]. Together with calls for SDM and structured patient education [7, 39], these findings emphasize communication strategies that directly address long‐term medication effects, psychosocial burden, and practical barriers.

Several limitations warrant consideration. First, the single‐center, cross‐sectional design and reliance on self‐reported data may limit generalizability and introduce recall bias. While the IBD‐KNOW was translated by experts for conceptual equivalence, its Chinese version awaits formal psychometric validation. To minimize respondent burden and anonymity‐related recall bias, detailed surgical history and educational attainment were not collected. Given that education is a known confounder for health literacy, its absence should be considered when interpreting IBD‐KNOW predictors. Additionally, “advanced therapies” were assessed as a broad category without distinguishing between biologics and small‐molecule therapies (SMTs), limiting the granularity of pharmacological preference data. Despite these constraints, the study highlights the multifaceted needs of patients with IBD and several avenues for improvement, including locally adapted digital education tools, longitudinal assessment of evolving preferences and needs, exploration of integrative approaches, and development of decision aids tailored to local healthcare constraints.

Future prospective studies evaluating the concordance between patient‐stated treatment preferences and actual therapy initiation in real‐world SDM settings would provide important insights into treatment selection dynamics in IBD care.

## Conflicts of Interest

The authors declare no conflicts of interest.

## Data Availability

The data that support the findings of this study are available from the corresponding author upon reasonable request.
